# Supplementary material for: Membrane manipulation by free fatty acids improves microbial plant polyphenol synthesis
Source: Nat Commun. 2023 Sep 12;14:5619. doi: 10.1038/s41467-023-40947-x (PMC10497605; doi:10.1038/s41467-023-40947-x)
Supplement: Supplementary file 1 — Supplementary Information [file 41467_2023_40947_MOESM1_ESM.docx]

**Membrane manipulation by free fatty acids improves microbial plant polyphenol synthesis**

*Supplementary Information*

Tharmasothirajan *et al.*


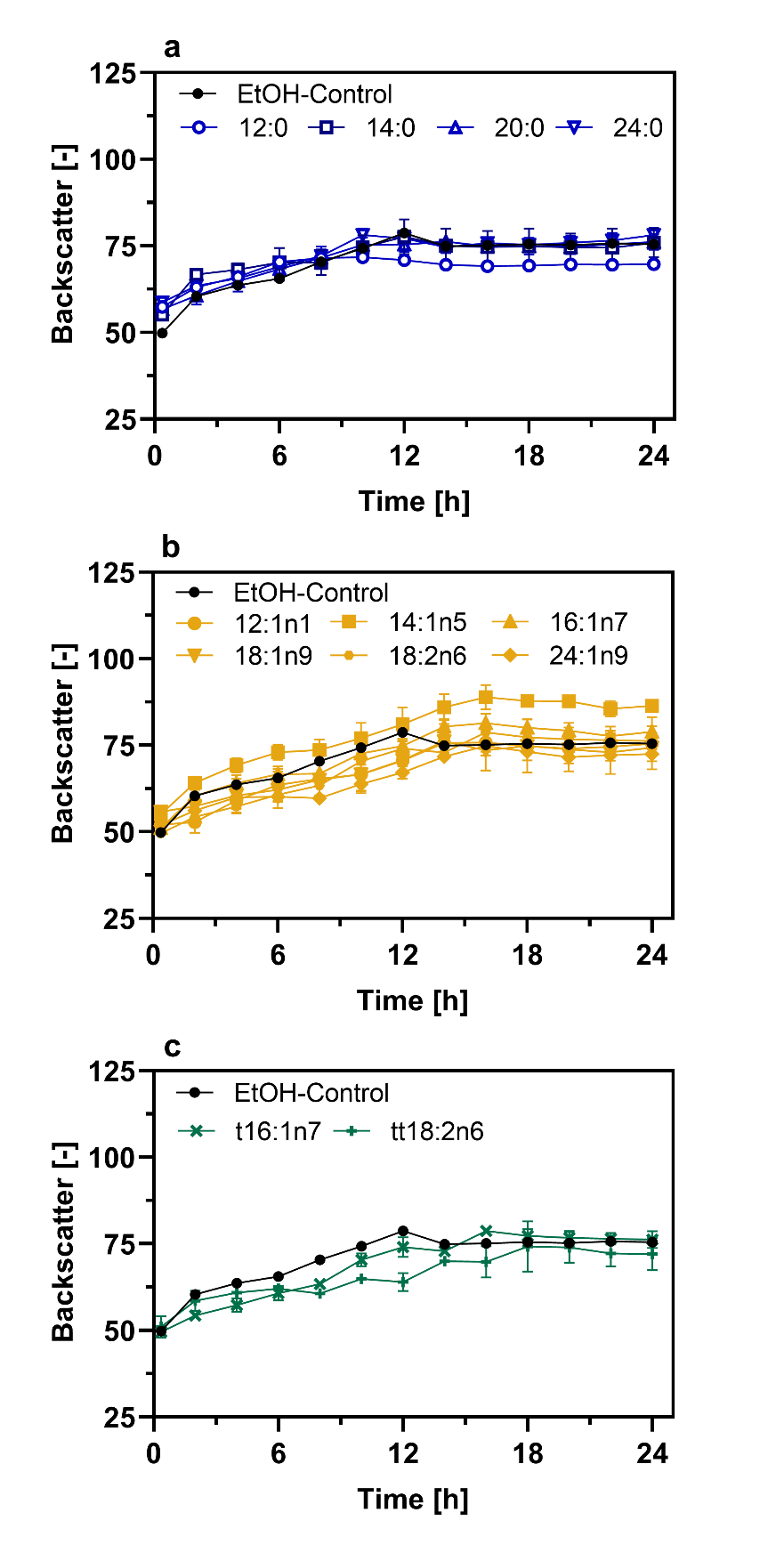


Supplementary Fig. 1 | Growth of *C. glutamicum*-RES1 on different FAs. BioLector cultivations in defined CGXII medium supplemented with 40 µM of a. saturated FAs b. unsaturated *cis*-FAs and c. unsaturated *trans*-FAs as sole carbon and energy source. Data represent average values and standard deviations of three biologically independent replicates (n=3). Source data are provided as a Source Data file.


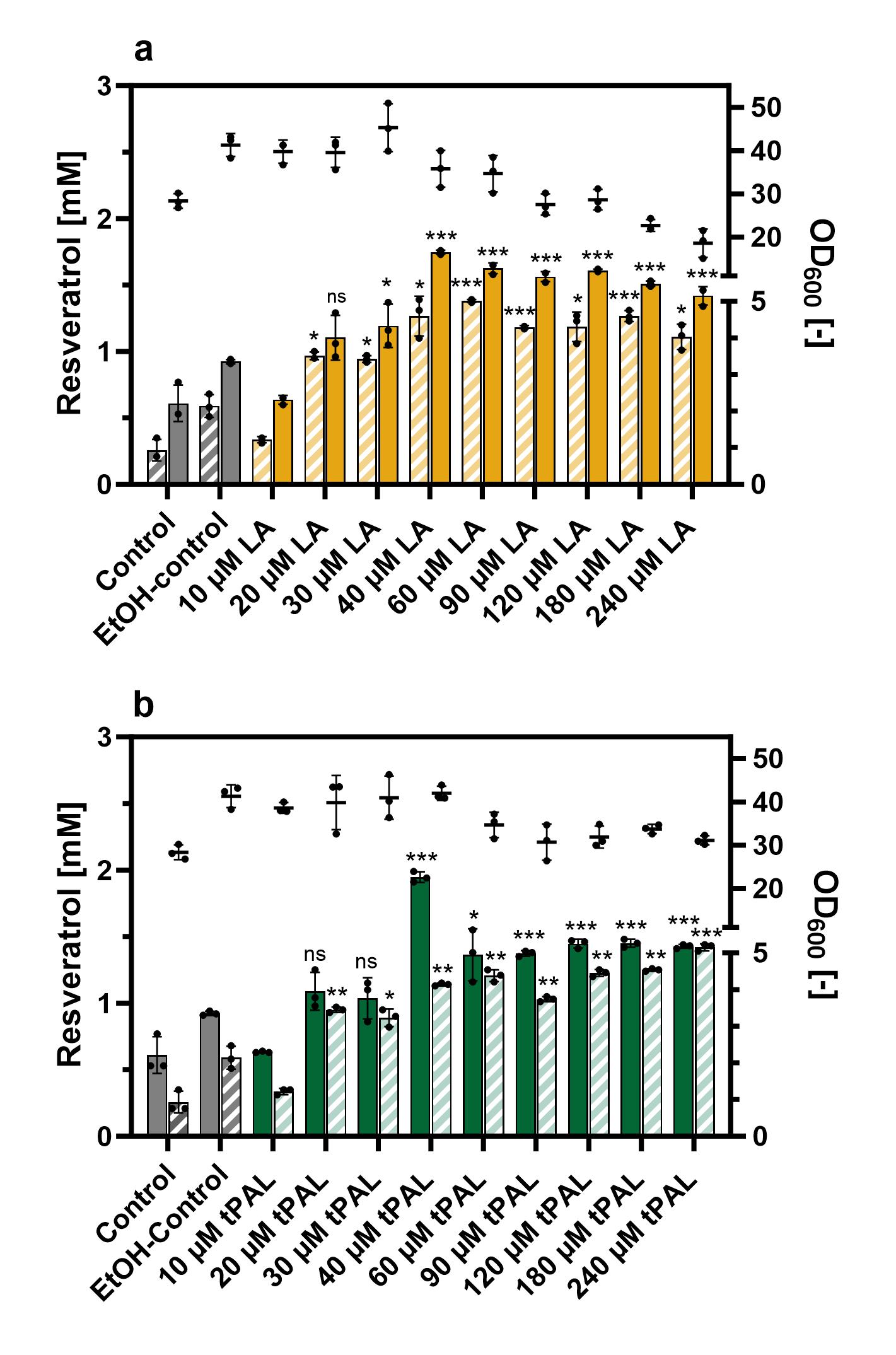


Supplementary Fig. 2 | Investigation of different concentrations of LA and tPAL for improved RES production. a Different concentrations (10, 20, 30, 40, 60, 90, 120, 240 µM) of LA and b tPAL were supplemented to determine the most beneficial FA concentration for RES production. Filled bars represent total RES, whereas striped bars represent extracellular RES. Data points represent the final OD_600_. Data represent average values and standard deviations of three biologically independent replicates (n=3). Statistical significance between the experimental groups and the EtOH-control for total RES and extracellular RES was assessed using a two-tailed unpaired Student’s *t* test (****p* < 0.001, ***p* < 0.01, **p* < 0.05, ns= not significant). Source data are provided as a Source Data file, including exact *p*-values.


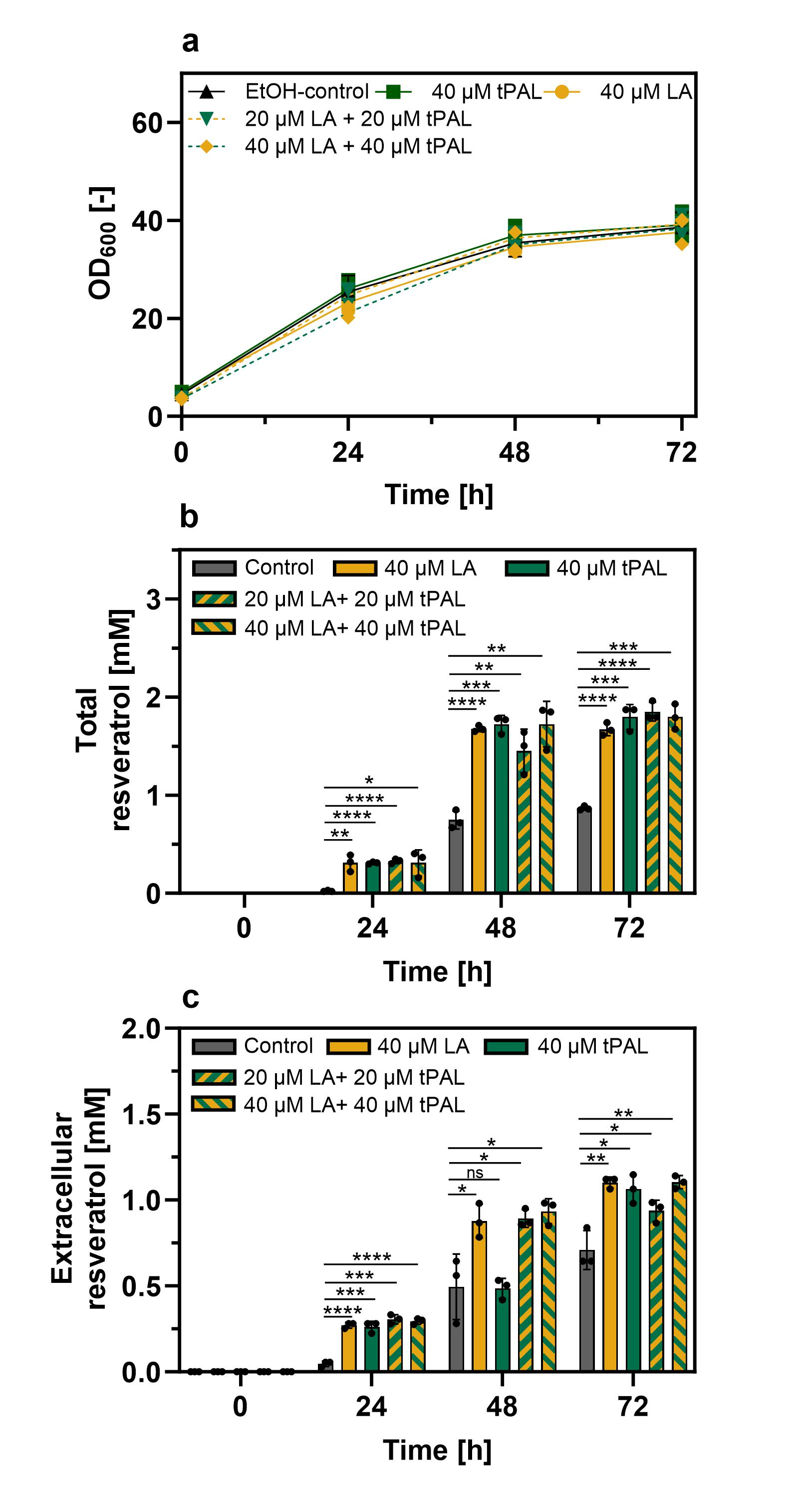


Supplementary Fig. 3 | RES production of *C. glutamicum*-RES1 in presence or absence of LA, tPAL and LA+tPAL mixtures. a Growth of *C. glutamicum-*RES1, b total RES and c extracellular RES during shake flask cultivations. 40 µM of LA (yellow), tPAL (green), 20 µM LA + 20 µM tPAL (yellow with green diagonal stripes), 40 µM LA + 40 µM tPAL (green with yellow diagonal stripes) were added to the cultures at the beginning of the respective cultivation. Data represent average values and standard deviations of three biologically independent replicates (n=3). Statistical significance was calculated by a two-tailed unpaired Student’s *t* test (*****p* < 0.0001, ****p* < 0.001, ***p* < 0.01, **p* < 0.05, ns= not significant). Source data are provided as a Source Data file, including exact *p*-values.

**

Supplementary Fig. 4 | Membrane fluidity of *C. glutamicum*-RES1 in presence of RES. Kinetic Laurdan measurements with stained *C. glutamicum*-RES1 cells at OD_600_ of 0.5. After 10 min, different RES concentration (0.13 mM, 0.25 mM, 0.5 mM, 0.75 mM) were added to the cells. The same volume of DMSO (1% (v v^-1^)) was added as a control to exclude possible interferences by increasing the volume. Data of each graph represent average values and standard deviations from three biologically independent replicates. Source data are provided as a Source Data file.

**Supplementary Fig. 5** l **Snapshots of *C. glutamicum* model membrane patches derived from MD simulations**. **a** Control lipid membrane composition (no additives), **b** Lipid membrane with added RES (purple) showing its partitioning to the membrane surface, **c** Lipid membrane with added RES and LA (orange). **d** Lipid membrane with added RES and tPAL (blue-green). Snapshots show all molecules except water molecules and ions, which are omitted for clarity. Colour code: dark grey POPG, light green DPPI, navy blue PODG, cyan (PO)MGDG, purple RES, orange LA, blue-green tPAL. Source data are provided as a Source Data file.


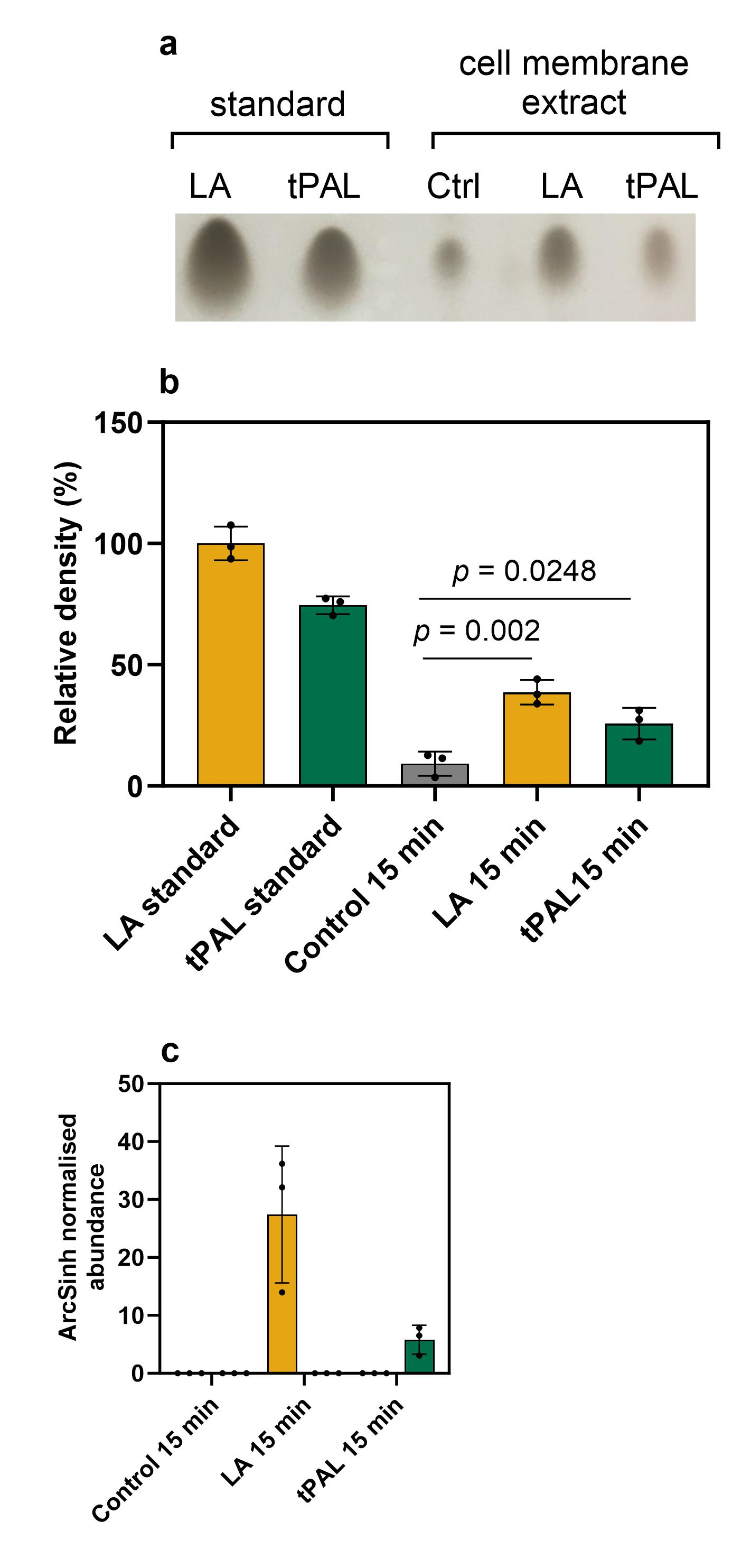


**Supplementary Fig. 6** **|** **Identification of LA and tPAL as free FAs in membrane extracts of *C. glutamicum-*RES1.** Cells were harvested 15 min after addition of either 40 μM LA (yellow), 40 μM tPAL (green) or without any FA supplementation. **a** Membrane lipids of these cells were extracted, separated by thin layer chromatography and stained with 10% CuSO_4_. **b** The relative densities of the FA spots were determined using ImageJ and values were normalized to LA standard (60 μM) **c** The presence of LA and tPAL in each of the three experimental conditions was investigated using HPLC-MS, and data was analyzed using Progenesis QI software. All data represents average values and standard deviation of three biologically independent replicates (n=3). Statistical significance was calculated by a two-tailed unpaired Student’s *t* test. Source data are provided as a Source Data file.


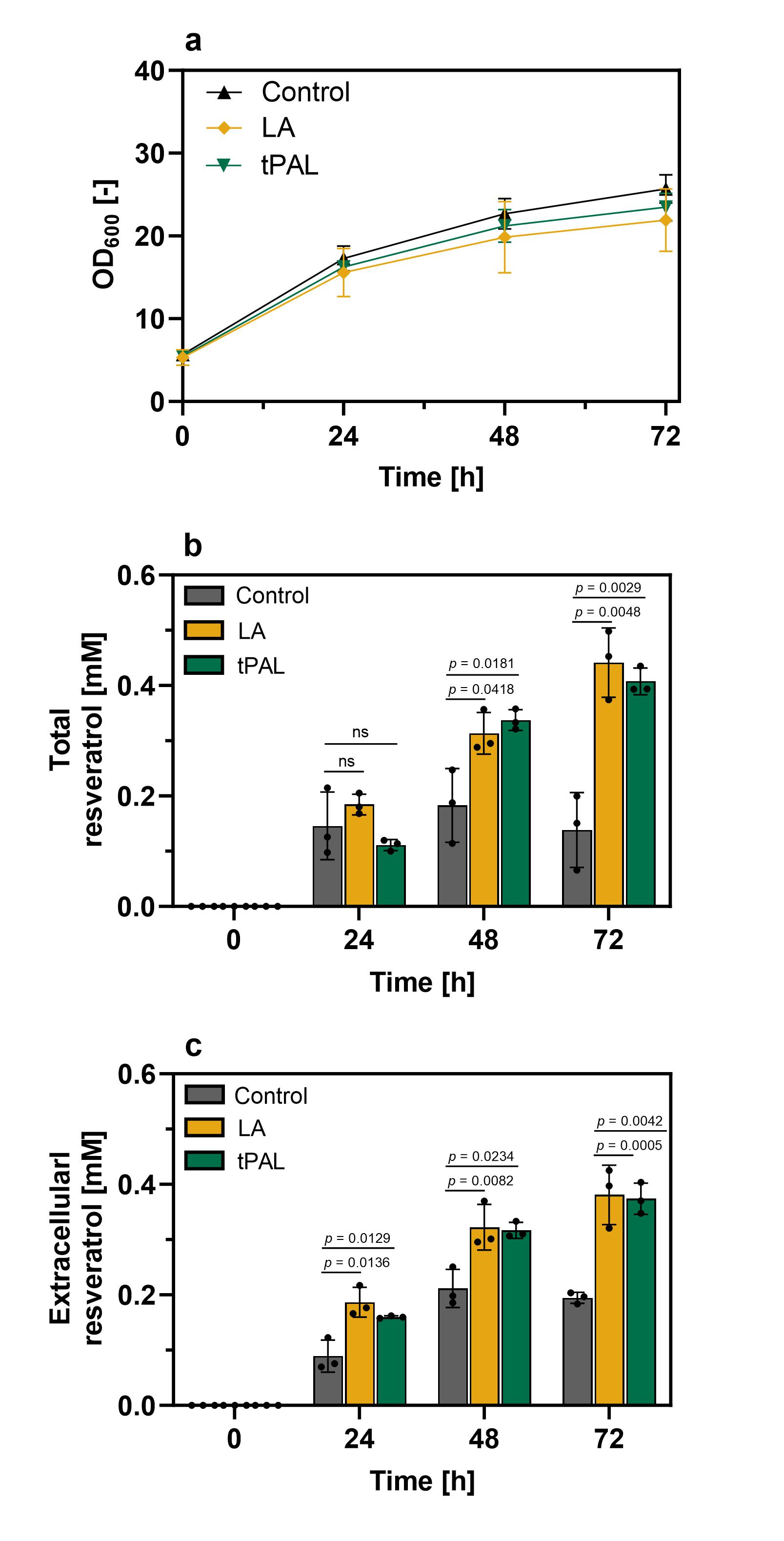


**Supplementary Fig. 7 | RES production from d-glucose in presence or absence of LA or tPAL using *C. glutamicum*-RES2. A** Growth of *C. glutamicum-*RES2, **b** total RES and **c** extracellular RES during shake flask cultivations. 40 µM of LA (yellow) or tPAL (green) were added to the cultures at the beginning of the respective cultivation. Data represent average values and standard deviation of three biologically independent replicates (n=3). Statistical significance was calculated by a two-tailed unpaired Student’s *t* test. Source data are provided as a Source Data file.


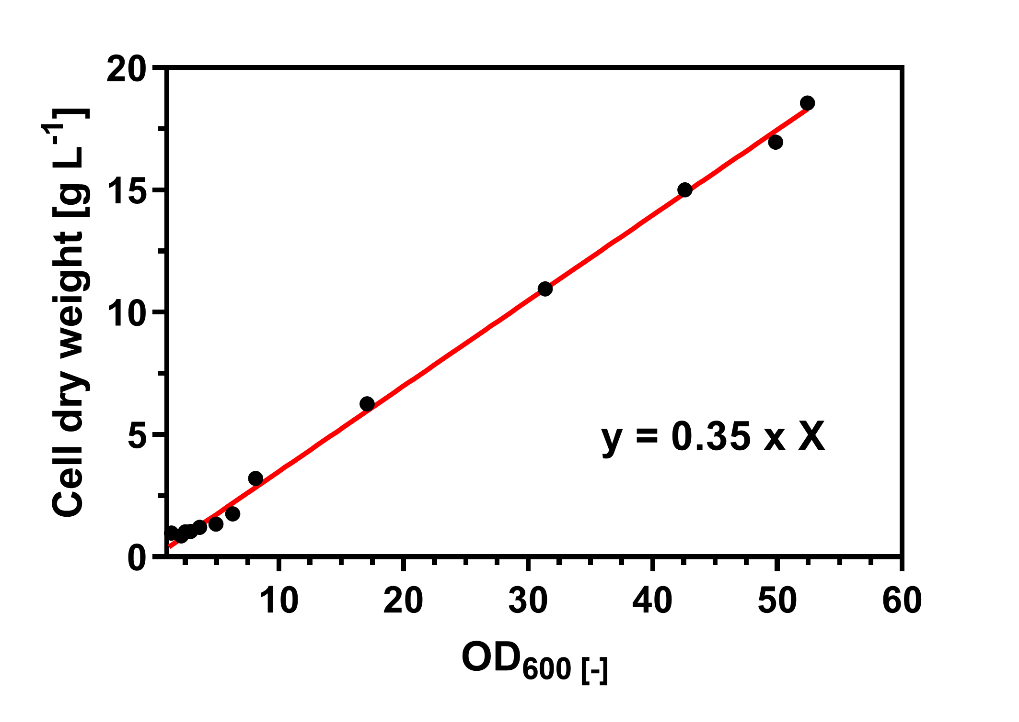


Supplementary Fig. 8 | Correlation between the optical density of *C. glutamicum* cultures at 600 nm (OD_600_) and cell dry weight. 0.35 g L^-1^ cell dry weight corresponds to an OD_600_ of 1. Source data are provided as a Source Data file.

**Supplementary Table 1 | Area compressibility of POPC bilayers.** Bilayers systems composed of 1352 lipids and 49256 water beads, in presence of 0, 10, or 50 mol% RES, simulated for 10 μs using standard settings as described in the Methods section. The block averaging method was used for the error estimation.

| POPC | POPC + 10 mol% RES | POPC + 50 mol% RES |
| --- | --- | --- |
| 69 ± 1 kT/nm^2^ | 66 ± 1 kT/nm^2^ | 67 ± 1 kT/nm^2^ |
