# Supplementary material for: Membrane manipulation by free fatty acids improves microbial plant polyphenol synthesis
Source: Nat Commun. 2023 Sep 12;14:5619. doi: 10.1038/s41467-023-40947-x (PMC10497605; doi:10.1038/s41467-023-40947-x)
Supplement: Supplementary file 3 — Description of Additional Supplementary Files [file 41467_2023_40947_MOESM3_ESM.pdf]

**Description of Additional Supplementary Files**

File Name: Supplementary  Movie 1
Description: Time course of the *C. glutamicum* model membrane simulation (0.5 µs trajectory) with added RES. Colour code: dark grey POPG, light green DPPI, navy blue PODG, cyan (PO)MGDG, purple RES.

File Name: Supplementary Movie 2
Description: Time course of the *C. glutamicum* model membrane simulation (0.5 µs trajectory) without RES. Colour code: dark grey POPG, light green DPPI, navy blue PODG, cyan (PO)MGDG.

File Name: Supplementary Movie 3
Description: Time course of the *C. glutamicum* model membrane simulation (0.5 µs trajectory) with added RES and LA. Colour code: dark grey POPG, light green DPPI, navy blue PODG, cyan (PO)MGDG, purple RES, orange LA.

File Name: Supplementary Movie 4
Description: Time course of the *C. glutamicum* model membrane simulation (0.5 µs trajectory) with added RES and LA. Colour code: dark grey POPG, light green DPPI, navy blue PODG, cyan (PO)MGDG. For more clarity, both RES and LA are not shown.
