# Supplementary material for: Membrane manipulation by free fatty acids improves microbial plant polyphenol synthesis
Source: Nat Commun. 2023 Sep 12;14:5619. doi: 10.1038/s41467-023-40947-x (PMC10497605; doi:10.1038/s41467-023-40947-x)
Supplement: Supplementary file 8 — Reporting Summary [file 41467_2023_40947_MOESM8_ESM.pdf]

nature portfolio

Corresponding author(s): Jan Marienhagen

Last updated by author(s): Nov 21, 2022

Reporting Summary

Nature Portfolio wishes to improve the reproducibility of the work that we publish. This form provides structure for consistency and transparency in reporting. For further information on Nature Portfolio policies, see our Editorial Policies and the Editorial Policy Checklist.

Statistics

For all statistical analyses, confirm that the following items are present in the figure legend, table legend, main text, or Methods section.

n/a	Confirmed	
☐	☒	The exact sample size (*n*) for each experimental group/condition, given as a discrete number and unit of measurement
☐	☒	A statement on whether measurements were taken from distinct samples or whether the same sample was measured repeatedly
☐	☒	The statistical test(s) used AND whether they are one- or two-sided<br>*Only common tests should be described solely by name; describe more complex techniques in the Methods section.*
☒	☐	A description of all covariates tested
☒	☐	A description of any assumptions or corrections, such as tests of normality and adjustment for multiple comparisons
☐	☒	A full description of the statistical parameters including central tendency (e.g. means) or other basic estimates (e.g. regression coefficient) AND variation (e.g. standard deviation) or associated estimates of uncertainty (e.g. confidence intervals)
☐	☒	For null hypothesis testing, the test statistic (e.g. *F*, *t*, *r*) with confidence intervals, effect sizes, degrees of freedom and *P* value noted<br>*Give P values as exact values whenever suitable.*
☒	☐	For Bayesian analysis, information on the choice of priors and Markov chain Monte Carlo settings
☒	☐	For hierarchical and complex designs, identification of the appropriate level for tests and full reporting of outcomes
☒	☐	Estimates of effect sizes (e.g. Cohen's *d*, Pearson's *r*), indicating how they were calculated

Our web collection on statistics for biologists contains articles on many of the points above.

Software and code

Policy information about availability of computer code

Data collection	OpenLab CDS 2.7 - liquid chromatography and gas chromatography data collection<br>MassLynx 4.2 - mass spectrometry data collection
Data analysis	BioLection 3.18.4.0 - to run BioLector microtiter plate cultivations and to analyze obtained data (biomass formation)<br>SPCImage 8.4 - calculation of fluorescence lifetime images<br>Progenesis QI software 2.6 - analysis of mass spectrometry data<br>GROMACS32 simulation package v2019 - used to run molecular dynamics simulations<br>VMD 1.9.3 - generation of snapshots from molecular dynamics simulations<br>GraphPad Prism 10 - statistical analysis

For manuscripts utilizing custom algorithms or software that are central to the research but not yet described in published literature, software must be made available to editors and reviewers. We strongly encourage code deposition in a community repository (e.g. GitHub). See the Nature Portfolio guidelines for submitting code & software for further information.

Data

Policy information about availability of data

All manuscripts must include a data availability statement. This statement should provide the following information, where applicable:

- Accession codes, unique identifiers, or web links for publicly available datasets
- A description of any restrictions on data availability
- For clinical datasets or third party data, please ensure that the statement adheres to our policy

The authors declare that the data supporting the findings of this study are available within the paper and its supplementary information file. A reporting summary for this article and source data are also provided with this paper. Additional data generated in this study can be made available upon request to the corresponding author.

Human research participants

Policy information about studies involving human research participants and Sex and Gender in Research.

Reporting on sex and gender	N/A
Population characteristics	N/A
Recruitment	N/A
Ethics oversight	N/A

Note that full information on the approval of the study protocol must also be provided in the manuscript.

Field-specific reporting

Please select the one below that is the best fit for your research. If you are not sure, read the appropriate sections before making your selection.

☒ Life sciences ☐ Behavioural & social sciences ☐ Ecological, evolutionary & environmental sciences

For a reference copy of the document with all sections, see nature.com/documents/nr-reporting-summary-flat.pdf

Life sciences study design

All studies must disclose on these points even when the disclosure is negative.

Sample size	No statistical methods were used to predetermine any sample size. Sample size was always based on experimental feasibility and sample availability.
Data exclusions	No data was excluded.
Replication	All cultivation experiments in absence or presence of fatty acids, and polyphenol quantifications were performed independently and in triplicates (stated in the text, figure legends and in the methods section). All attempts of replication were successful.
Randomization	Randomization was not necessary - we knew the genetic constitution of all production strains (Sanger sequencing). All subsequent characterizations were performed in triplicates. Covariates do not apply, since all cells of all cultures are in the same growth state and all environmental conditions are the same for all samples at all times.
Blinding	Blinding does not apply. Knowledge of the e.g. identity of the respective fatty acid supplements used or the time point at which a sample was taken is essential to assign the experimentally determined effects.

Reporting for specific materials, systems and methods

We require information from authors about some types of materials, experimental systems and methods used in many studies. Here, indicate whether each material, system or method listed is relevant to your study. If you are not sure if a list item applies to your research, read the appropriate section before selecting a response.

Materials & experimental systems

n/a	Involved in the study
☒	☐ Antibodies
☒	☐ Eukaryotic cell lines
☒	☐ Palaeontology and archaeology
☒	☐ Animals and other organisms
☒	☐ Clinical data
☒	☐ Dual use research of concern

Methods

n/a	Involved in the study
☒	☐ ChIP-seq
☒	☐ Flow cytometry
☒	☐ MRI-based neuroimaging
